# Editorial: Updating long COVID: mechanisms, risk factors, and treatment

**DOI:** 10.3389/fnhum.2024.1490242

**Published:** 2024-09-19

**Authors:** César Fernández-de-las-Peñas, Lars Arendt-Nielsen

**Affiliations:** ^1^Department of Physical Therapy, Occupational Therapy, Physical Medicine and Rehabilitation, Universidad Rey Juan Carlos, Madrid, Spain; ^2^Department of Health Science and Technology, Center for Neuroplasticity and Pain, School of Medicine, Aalborg University, Aalborg, Denmark; ^3^Department of Gastroenterology and Hepatology, Mech-Sense, Clinical Institute, Aalborg University Hospital, Aalborg, Denmark; ^4^Steno Diabetes Center North Denmark, Clinical Institute, Aalborg University Hospital, Aalborg, Denmark

**Keywords:** COVID-19, post-COVID, long-COVID symptoms, mechanism, risk factors

## Introduction

The rapid spread of the coronavirus disease, 2019 (COVID-19) has provoked the most unprecedent sanitary crisis of this century leading to up to 776 million confirmed cases and more than 7 million deaths worldwide (WHO, 2023). In fact, the Severe Acute Respiratory Syndrome Coronavirus 2 (SARS-CoV-2), the pathogen responsible of COVID-19, has become one of the most investigated virus due to a total explosion of research thanks to the publication of thousands and thousands of papers in a relatively small period of time.

Extensive research aiming to decrease the severity and mortality of COVID-19 has been published. For instance, administration of antivirals at an early stage of the acute COVID-19 phase has shown to decrease mortality rate, hospitalization stay, and COVID-19 severity (Zur et al., 2024). Similarly, the development of COVID-19 vaccines has been one of the most important advances in the fight against SARS-CoV-2. Thus, vaccines have demonstrated to be effective for reducing the risk of severe COVID-19 and also associated mortality (Dinagde et al., 2024); however, vaccines have not been effective for preventing SARS-CoV-2 contagion (Wang et al., 2022). In fact, SARS-CoV-2 trophism has resulted in the appearance of several variants in a short period of time (Jagst et al., 2024). The worldwide spread of new variants of concern has led to reinfections (Sciscent et al., 2021).

Albeit all the progress and efforts done for fighting against SARS-CoV-2, another growing healthcare problem derived from COVID-19 is the presence of long-lasting or persistent symptoms once the acute infection has passed. The presence of persistent long-lasting symptoms after the acute infection has received several and heterogenous names from the beginning of the pandemic (Yang et al., 2024), being long-COVID (Fernández-de-las-Peñas, 2022) or post-COVID-19 condition (Soriano et al., 2022) the terms most accepted. Some meta-analyses have reported that up to 25–30% of COVID-19 survivors exhibit post-COVID symptomatology one (Chen et al., 2022; Han et al., 2022) and two (Fernández-de-las-Peñas et al., 2024; Rahmati et al., 2023) years after an acute SARS-CoV-2 infection. Further, a recent meta-analysis estimated a pooled worldwide prevalence of long-COVID of 41.8% (95% CI 39.7–43.9%) (Sk Abd Razak et al., 2024), although this rate is based on studies including COVID-19 survivors infected during the first year of the pandemic, mostly with the historical strain and Delta variable. In fact, it has been found that the average direct medical costs of a patient with post-COVID symptoms ranges from US $1,264 to 79,315 (Faramarzi et al., 2024).

Nevertheless, several gaps in different aspects of post-COVID-19 condition exist. The aim of the Research Topic “*Updating long COVID: mechanisms, risk factors, and treatment*” published in Frontiers has focused on several aspects of post-COVID-19 condition, a topic of emerging relevance due to the presence of millions of “long-haulers” worldwide. In this Editorial, we discuss the following topics: 1, mechanisms underlying post-COVID-19 condition; 2, clustering of post-COVID symptoms; and 3, risk factors of post-COVID-19 condition.

## Mechanisms underlying post-COVID-19 condition

Several hypotheses such as viral persistence, long-lasting inflammation, endothelial dysfunction, alteration in microbiota, immune dysregulation/autoimmunity or autonomic dysfunction have been proposed for explaining the presence of post-COVID symptoms (Fernández-de-las-Peñas et al., 2023). However, the heterogeneity of post-COVID symptoms suggests an association with different predominant pathophysiological mechanism operating on each symptom. In this Research Topic, papers investigated different mechanisms potentially associated with post-COVID fatigue and neurocognitive symptoms.

da Silva et al. investigated the presence of autonomic dysfunction in individuals with post-COVID-19 condition and reported a long-lasting sympathetic predominance during the head-up tilt test. The presence of an autonomic dysfunction could explain general post-COVID symptomatology such as fatigue or even post-exertional malaise, which is experienced by almost 50% of long-haulers (Pagen et al., 2023). In a pilot study, Hofmann et al. found that the presence of post-COVID fatigue was associated with a reduction of superoxide anion (O·-2) formation, suggesting that oxidative stress induces chronic fatigue-like symptoms in these patients. In such scenario, a long-lasting oxidative stress state may also explain the development of post-exertional malaise.

Yao et al. observed a reduced overall brain activity and a rearrangement of several brain functional networks in a small sample of individuals who had been infected with COVID-19. This study did not include subjects with post-COVID-19 condition since evaluations were conducted 28 days after the main infection, but authors proposed that the presence of brain abnormalities soon after an acute SARS-CoV-2 infection could be related to damage of the nervous system by the virus explaining the potential development of post-COVID neurocognitive symptomatology (Yao et al.). Thus, the narrative review by Zhao et al. discussed the mechanisms of neurodegenerative post-COVID diseases by examining the pathways of central nervous system infection by SARS-CoV-2. Current evidence supports that chronic inflammation and abnormal immune responses can lead to neuronal damage and long-term post-COVID neurocognitive symptomatology (Zhao et al.).

## Clusters and post-COVID symptomatology

Due to the heterogeneity of post-COVID symptomatology, different attempts to identify subgroups (cluster) of patients. In fact, two different types of clusters by grouping of symptoms (e.g., neurological, cardiorespiratory or systemic/inflammatory cluster) or by prognosis (e.g., improved, non-improved, stable) have been identified (Kuodi et al., 2023). In the current Research Topic, both types of clustering were investigated.

Chen et al. identified the clinical features of three clusters based on the evolution or prognosis of symptoms based on the 3-month change in symptom number: remittent, persistent, or incident. These authors found that the incident phenotype was younger, had lower hospitalization rate but a greater number of post-COVID symptoms associated with systemic corticosteroid administration during the acute SARS-CoV-2 infection than the persistent or remittent phenotypes (Chen et al.).

Núñez et al. classified groups of patients by their different type of post-COVID symptoms and identified respiratory and neurocognitive symptoms clusters. Thus, the study by Carmona-Cervelló et al. found a heterogeneous battery of neurocognitive post-COVID symptoms associated with the presence of deficits in executive functions. This study suggests that one post-COVID symptom cluster e.g., neurocognitive, can also present different associated symptomatology (Carmona-Cervelló et al.). Finally, Fernández-de-las-Peñas et al. investigated the longitudinal evolution of three post-COVID neurocognitive symptoms (e.g., brain fog, memory loss, and concentration loss) during the first two tears after the infection and, overall, they found a decreasing trend as expressed by exponential bar plots.

## Risk factors

Different meta-analyses identifying potential risk factor associated with post-COVID symptoms have been published (Tsampasian et al., 2023; Luo et al., 2024). These meta-analyses found that female sex, older age, severe COVID-19, previous medical comorbidities, longer hospitalization stay, and high body mass index were associated with a higher risk of post-COVID-19 condition (Tsampasian et al., 2023; Luo et al., 2024).

In the current Research Topic, a Colombian study reported that female sex, severe COVID-19 (requirement of mechanical ventilation), some medical co-morbidities e.g., such as Chronic Obstructive Pulmonary Disease (COPD) or rheumatic disease and longer hospitalization stay, but not older age, were associated with a higher risk of developing long-COVID (Martínez-Ayala et al.). A study conducted in Brazil also found that female sex and medical co-morbidities such as hypertension were also a risk factor associated with the presence of long-COVID (Eduvirgem et al.). This Brazilian study identified that suffering from a higher number of COVID-10 associated-onset symptoms and being infected with the historical strain during the first wave of the pandemic were factors associated with a higher risk of long-COVID (Eduvirgem et al.). It seems that female sex is the risk factor most associated with the development of post-COVID-19 condition, whereas other factors, e.g., older age, severe COVID-19, or longer stay at hospital, depend on the study design. Finally, two papers published in the current Research Topic observed not only female sex as a risk factor for post-COVID symptoms but also that clinical post-COVID symptoms are experienced differently between females and males (Marcilla-Toribio et al.) and that women showed different serological biomarkers, e.g., lower ferritin and procalcitonin levels but higher TNF levels at the acute COVID-19 phase than males (Delfino et al.). Current and previous findings will support that healthcare systems should consider long-COVID from a sex perspective at all.

In conclusion, this Research Topic has permitted to advance in current knowledge of long-COVID by providing further evidence on some underlying mechanisms (such as autonomic dysfunction, hyper-oxidative stress, chronic brain inflammation, and abnormal immune responses), confirming the presence of different post-COVID clusters and also confirming that female sex is probably the risk factor most predominantly associated with the development of this condition.

As Guest Editors of this Research Topic, we would like to thank to the reviewers for their comments, to all contributors for their valuable papers, and to Frontiers Research Topic staff for their collective support and assistance during this process.

## References

[B1] Chen C. Haupert S. R. Zimmermann L. Shi X. Fritsche L. G. Mukherjee B.. (2022). Global prevalence of post COVID-19 condition or long COVID: a meta-analysis and systematic review. J. Infect. Dis. 226, 1593–1607. 10.1093/infdis/jiac136 35429399 PMC9047189

[B2] Dinagde D. D. Degefa B. D. Kitil G. W. Feyisa G. T. Marami S. N. (2024). SARS-CoV-2 infection after COVID-19 vaccinations among vaccinated individuals, prevention rate of COVID-19 vaccination: a systematic review and meta-analysis. Heliyon 10:e30609. 10.1016/j.heliyon.2024.e30609 38737290 PMC11087980

[B3] Faramarzi A. Norouzi S. Dehdarirad H. Aghlmand S. Yusefzadeh H. Javan-Noughabi J.. (2024). The global economic burden of COVID-19 disease: a comprehensive systematic review and meta-analysis. Syst. Rev. 13:68. 10.1186/s13643-024-02476-6 38365735 PMC10870589

[B4] Fernández-de-las-Peñas C. (2022). Long COVID: current definition. Infection 50, 285–286. 10.1007/s15010-021-01696-5 34519974 PMC8438555

[B5] Fernández-de-las-Peñas C. Notarte K. I. Macasaet R. Velasco J. V. Catahay J. A. Therese Ver A.. (2024). Persistence of post-COVID symptoms in the general population two years after SARS-CoV-2 infection: a systematic review and meta-analysis. J. Infect. 88, 77–88. 10.1016/j.jinf.2023.12.004 38101521

[B6] Fernández-de-las-Peñas C. Raveendran A. V. Giordano R. Arendt-Nielsen L. (2023). Long COVID or post-COVID-19 condition: past, present and future research directions. Microorganisms 11:2959. 10.3390/microorganisms11122959 38138102 PMC10745830

[B7] Han Q. Zheng B. Daines L. Sheikh A. (2022). Long-term sequelae of COVID-19: a systematic review and meta-analysis of one-year follow-up studies on post-COVID symptoms. Pathogens 11:269. 10.3390/pathogens11020269 35215212 PMC8875269

[B8] Jagst M. Pottkämper L. Gömer A. Pitarokoili K. Steinmann E. (2024). Neuroinvasion and neurotropism of severe acute respiratory syndrome coronavirus 2 infection. Curr. Opin. Microbiol. 79:102474. 10.1016/j.mib.2024.102474 38615394

[B9] Kuodi P. Gorelik Y. Gausi B. Bernstine T. Edelstein M. (2023). Characterization of post-COVID syndromes by symptom cluster and time period up to 12 months post-infection: a systematic review and meta-analysis. Int. J. Infect. Dis. 134, 1–7. 10.1016/j.ijid.2023.05.003 37150350

[B10] Luo D. Mei B. Wang P. Li X. Chen X. Wei G.. (2024). Prevalence and risk factors for persistent symptoms after COVID-19: a systematic review and meta-analysis. Clin. Microbiol. Infect. 30, 328–335. 10.1016/j.cmi.2023.10.016c 37866679

[B11] Pagen D. M. E. Van Herck M. van Bilsen C. J. A. Brinkhues S. Konings K. den Heijer C. D. J.. (2023). High proportions of post-exertional malaise and orthostatic intolerance in people living with post-COVID-19 condition: The PRIME post-COVID study. Front. Med. 10:1292446. 10.3389/fmed.2023.1292446 38162880 PMC10757844

[B12] Rahmati M. Udeh R. Yon D. K. Lee S. W. Dolja-Gore X. McEVoy M.. (2023). A systematic review and meta-analysis of long-term sequelae of COVID-19 2-year after SARS-CoV-2 infection: a call to action for neurological, physical, and psychological sciences. J. Med. Virol. 95:e28852. 10.1002/jmv.28852 37288652

[B13] Sciscent B. Y. Eisele C. D. Ho L. King S. D. Jain R. Golamari R. R.. (2021). COVID-19 reinfection: the role of natural immunity, vaccines, and variants. J. Commun. Hosp. Intern. Med. Perspect. 11, 733–739. 10.1080/20009666.2021.1974665 34804382 PMC8604456

[B14] Sk Abd Razak R. Ismail A. Abdul Aziz A. F. Suddin L. S. Azzeri A. Sha'ari N. I. (2024). Post-COVID syndrome prevalence: a systematic review and meta-analysis. BMC Public Health 24:1785. 10.1186/s12889-024-19264-5 38965510 PMC11223303

[B15] Soriano J. B. Murthy S. Marshall J. C. Relan P. Diaz J. V. WHO Clinical Case Definition Working Group on Post-COVID-9 Condition (2022). A clinical case definition of post-COVID-19 condition by a Delphi consensus. Lancet Infect Dis. 22, e102–e107. 10.1016/S1473-3099(21)00703-9 34951953 PMC8691845

[B16] Tsampasian V. Elghazaly H. Chattopadhyay R. Debski M. Naing T. K. P. Garg P.. (2023). Risk factors associated with post-COVID-19 condition: A systematic review and meta-analysis. JAMA Int. Med. 183, 566–580. 10.1001/jamainternmed.2023.0750 36951832 PMC10037203

[B17] Wang K. Wang L. Li M. Xie B. He L. Wang M.. (2022). Real-word effectiveness of Global COVID-19 vaccines against SARS-CoV-2 variants: a systematic review and meta-analysis. Front. Med. 9:820544. 10.3389/fmed.2022.820544 35665358 PMC9160927

[B18] WHO (2023). WHO Coronavirus (COVID-19) Dashboard. Available at: https://covid19.who.int/ (accessed September 1, 2024).

[B19] Yang J. Markus K. Andersen K. M. Rudolph A. E. McGrath L. J. Nguyen J. L.. (2024). Definition and measurement of post-COVID-19 conditions in real-world practice: a global systematic literature review. BMJ Open 14:e077886. 10.1136/bmjopen-2023-077886 38233057 PMC10806676

[B20] Zur M. Peselev T. Yanko S. Rotshild V. Matok I. (2024). Efficacy and safety of antiviral treatments for symptomatic COVID-19 outpatients: systematic review and network meta-analysis. Antiviral Res. 221:105768. 10.1016/j.antiviral.2023.105768 38056602

